# Artemisia absinthium: burning plant!

**DOI:** 10.11604/pamj.2016.23.10.8605

**Published:** 2016-01-22

**Authors:** Najia Ilham El Makrini, Badredine Hassam

**Affiliations:** 1 Department of Dermatology, Ibn Sina Hospital, Rabat, Morocco

**Keywords:** Artemisia absinthium, phytodermatose, plant

## Image in medicine

Phytodermatoses are skin lesions secondary to prolonged contact with certain herbs. Many plants have been described responsible for this type of incident. Mrs. H., 50 years old, without pathological history, presented at the consultation for a sharp pain in the face, without any functional sign. The questioning found the application of a poultice, advised by a neighbor, containing the “Artemisia absinthium” to reduce wrinkles crow's feet and glabella. Dermatological examination revealed a dry and delicate erythema intersting cheeks and forehead without blistering, corresponding to a first degree burn. The care provided helped a favorable evolution in few days. The “Artemisia absinthium”, also called “green fairy” is a perennial plant with ubiquitous distribution in waste and dry places, on rocky slopes, in roadsides and fields. It is native to Europe, Northern and Central Asia as well as in North Africa. Leaves, deeply cut, are silver, oval, embossed, velvety below and toothed. Absinthe is gently used in herbal medicine for her virtues tonic, antispasmodic, antipyretic, anthelmintic, stimulating … However, this plant may contain toxic agents (such as thujone, malic acid, alcohol …) responsible for adverse reactions. In our case, use for cosmetic purposes has caused redness and sensitivity of the face, causing a chemical burn of the first degree. There is a phytodermatose never described to our knowledge.

**Figure 1:**
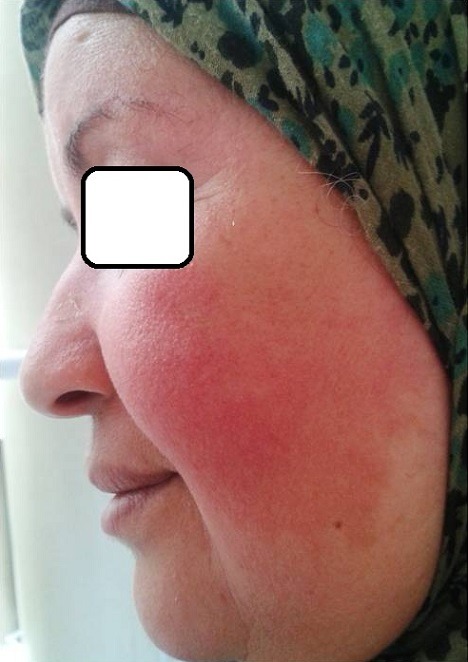
Erythema intersting cheeks and forehead

